# Development and Assessment of a Gastroscopy Electronic Learning System for Primary Learners: Randomized Controlled Trial

**DOI:** 10.2196/16233

**Published:** 2020-03-23

**Authors:** Shuang Li, Guoqing Li, Ying Liu, Wanying Xu, Ningning Yang, Haoyuan Chen, Ning Li, Kunpeng Luo, Shizhu Jin

**Affiliations:** 1 Department of Gastroenterology and Hepatology The Second Affiliated Hospital of Harbin Medical University Harbin China

**Keywords:** e-learning, gastroscopy, endoscopy, randomized controlled trial

## Abstract

**Background:**

Endoscopic examination is a popular and routine procedure for the diagnosis and treatment of gastrointestinal (GI) diseases. Skilled endoscopists are in great demand in clinical practice, but the training process for beginners to become endoscopy specialists is fairly long. Convenience and a self-paced, learner-centered approach make electronic learning (e-learning) an excellent instructional prospect.

**Objective:**

This study aimed to develop and apply an e-learning system in gastroscopy teaching and learning and to evaluate its effectiveness and user satisfaction.

**Methods:**

The e-learning software *Gastroscope Roaming System* was developed for primary learners. The system simulates the real structure of the upper gastrointestinal (UGI) tract to teach the main characteristics of gastroscopy under both normal conditions and conditions of common UGI tract diseases. A randomized controlled trial was conducted. Participants were randomly allocated to an e-learning group (EG)or a non–e-learning control group after a pretest. On completing the training, participants undertook a posttest and gastroscopy examination. In addition, the EG completed a satisfaction questionnaire.

**Results:**

Of the 44 volunteers, 41 (93%) completed the gastroscopy learning and testing components. No significant pretest differences were found between the intervention and control groups (mean 50.86, SD 6.12 vs mean 50.76, SD 6.88; *P*=.96). After 1 month of learning, the EG’s posttest scores were higher (mean 83.70, SD 5.99 vs mean 78.76, SD 7.58; *P*=.03) and improved more (*P*=.01) than those of the control group, with better performance in the gastroscopy examination (mean 91.05, SD 4.58 vs mean 84.38, SD 5.19; *P*<.001). Overall, 85% (17/20) of the participants were satisfied with the e-learning system, and 95% (19/20) of the participants considered it successful.

**Conclusions:**

E-learning is an effective educational strategy for primary learners to acquire skills in gastroscopy examination and endoscopic imaging of the GI tract.

**Trial Registration:**

Chinese Clinical Trial Registry ChiCTR-IOR-17013091; http://www.chictr.org.cn/showproj.aspx?proj=22142

## Introduction

### Background

With the development of digestive endoscopy technology, gastrointestinal (GI) diseases are increasingly treatable by digestive endoscopy. Skilled and experienced endoscopists are needed in clinical practice worldwide, yet the training process for primary learners is difficult and time consuming. Hence, educating endoscopists has become a global challenge in medical education.

Traditionally, experienced endoscopists have played an important role in training primary learners, and primary learners have worked with their tutors to practice endoscopy on patients [1]. It is common for primary learners to feel nervous and anxious and to be unable to obtain satisfactory cooperation from their patients. In addition, patients may refuse to permit primary learners to practice GI endoscopy on them. Traditional clinical training may fail to provide adequate information on multiple needs and cognitive deficits. Endoscopic simulators, including ex vivo animal tissue models, live animal models, mechanical models, and virtual reality computer simulators [2,3], have been widely used in academic practice. However, problems remain with regard to the high cost of teaching, limited resource space, and small audience, and primary learners can only study the structure of the upper gastrointestinal (UGI) tract and cannot further study the endoscopic manifestations of UGI diseases. Thus, improving the instructional programs and models for primary learners is a key issue in endoscopy training.

Many institutes are pioneering electronic learning (e-learning) methods as a cost-effective alternative to traditional methods [4,5]. E-learning is a well-established approach to learning via electronic-/computer-based, mostly Web-based, programs [6]. The technology is based on the use of the internet to deliver a broad array of educational materials and training procedures to enhance knowledge and performance [7,8]. Numerous researchers have described the use of e-learning in medical education [9-18]. Compared with traditional teaching environments, e-learning has several advantages, such as asynchrony, cost savings, individualized learning, greater accessibility, greater ease of distribution, and up-to-date content, which may overcome the difficulties and dilemmas experienced during the early phases of endoscopic training [19-22]. Learners may be attracted to e-learning because it centers them in the learning procedure, in contrast to their role as passive recipients in traditional training methods [23]. Learners enrolled in an e-learning program can choose the content that they access, the sequence in which it is studied, and the space used for learning based on their individual experience and personal learning objectives without time or space limitations [24,25].

### Aim of This Study

In this context, we designed and developed the Gastroscope Roaming System (GRS) to create, assess, and implement an integrated e-learning gastroscopic education package for primary learners. The system focuses on teaching the principal characteristics of gastroscopy examination of the human UGI under normal conditions and under conditions of routine UGI diseases. We aimed to (1) develop a gastroscopy e-learning system for primary learners, (2) evaluate the efficacy and effectiveness of the system, and (3) assess the satisfaction of primary learners.

## Methods

### Development and Description of the Electronic Learning System

We collaborated with the Harbin University of Science and Technology to develop the GRS. The system was built to be bilingual in Chinese and English; the language can be freely switched on the log-in interface, and the system can be used by primary learners of gastroscopy in China and worldwide. First, the user interface of the system was designed and created. A 3-dimensional model of the UGI tract, including the esophagus, stomach, and duodenum, was obtained after a review of various data sources (mainly pictures and videos of gastroscopy examinations from the Digestive Endoscopy Center of the Second Affiliated Hospital of Harbin Medical University [HMU]) and was modeled by Unity 3D, Autodesk Maya, Zbrush, and Substance Painter software. Then, the 3-dimensional model was transformed into an operation scene, and the physiological function was simulated and rendered (Adobe Photoshop software for material design and production and Maya software for animation). The C++ programming language was used to design the roaming, interactive, and cognitive functions of the digestive organ structure and to realize the camera placement, path switching, and perspective switching. In addition, embedded links and demonstrations of lesion cases were created. Currently, the program requires a specified account number and password for a user to log in.

**Figure 1:**
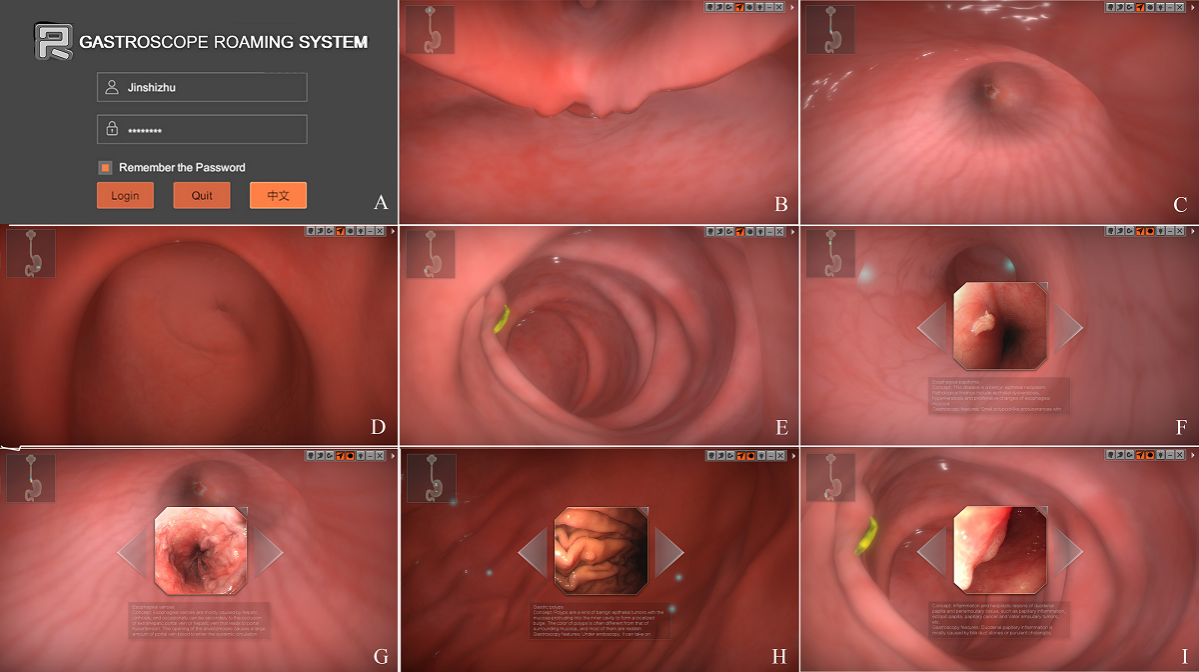
Gastroscope Roaming System’s software screen captures. (A) Software system log-in interface. (B) Normal esophageal entrance screenshot. (C) Normal cardia entrance screenshot. (D) Normal pyloric entrance screenshot. (E) Normal duodenal papilla screenshot. (F-I) Screenshots of common diseases in different parts of the disease learning state, and the disease description and gastroscopic characteristics are given below each image.

The two GRS modules were designed for primary learners to gain knowledge of the UGI anatomic structure, relevant endoscopy images, and protocol requirements and to improve their confidence. Module A contains normal human anatomic structures, with anatomic color illustrations from the pharyngeal portion to the descending part of the duodenum of the human UGI. Module B contains UGI images showing both normal conditions and conditions of routine UGI diseases. The software can also simulate UGI endoscopy and the route of the gastroscopy lens. The incidences of typical diseases along the endoscopic examination path are labeled based on clinical images of these diseases. Dozens of common UGI diseases are included for learning purposes. There are corresponding bilingual explanations under the images, including disease characteristics and endoscopic manifestations. Screenshots are presented in Figure 1.

### Participant Recruitment and Implementation Strategy

We issued an experimental information notice to recruit participants in the hospital. The 44 volunteers were all master degree students majoring in gastroenterology and hepatology at the Second Affiliated Hospital of HMU who had signed a consent form to participate in this trial. The teachers at the endoscopy center verified the basic information of each volunteer and played a key supervisory role in the entire process of learning and evaluation. All participants had acquired basic GI theoretical knowledge in their previous years of study, but because the GRS was designed for primary learners, the participants could not perform a gastroscopy and confirmed that they had not yet received systematic professional education in gastroscopy. In addition, they needed access to a procedure on their own computers to operate the sample version of the e-learning system. All participants completed a pretest questionnaire; those who did not complete the pretest or did too well in the pretest (correct rating >90% or higher) were excluded because the e-learning was designed for beginners who had not previously mastered sufficient endoscopy knowledge [26].

**Figure 2:**
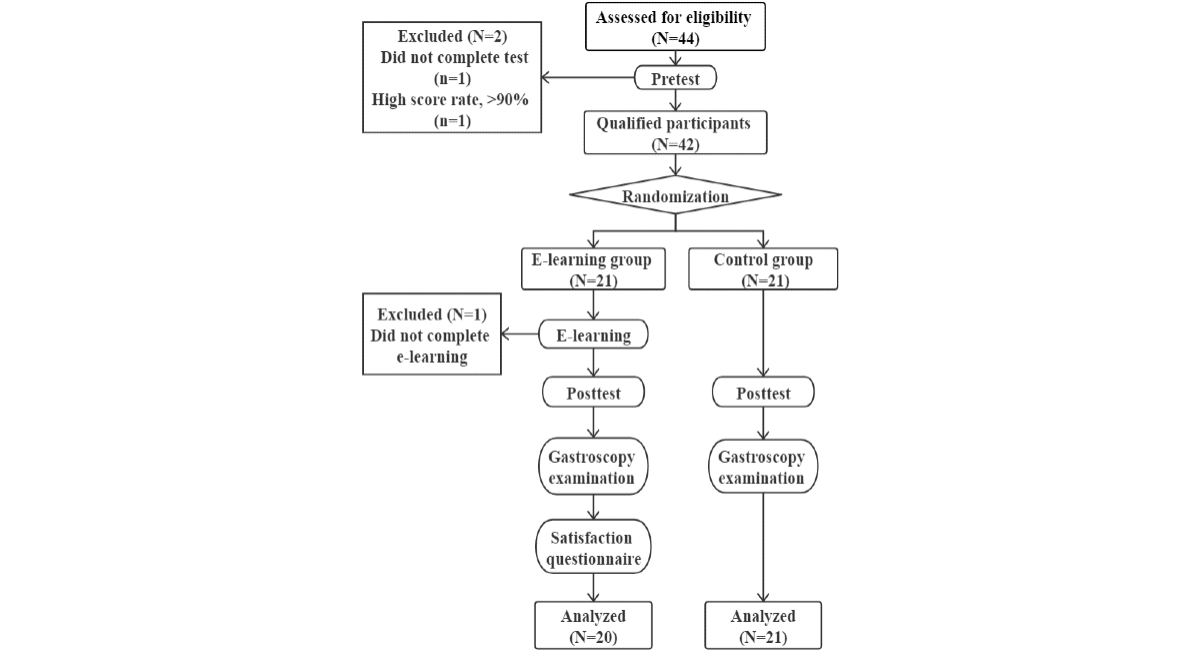
Flow diagram showing the details of the participant enrollment, randomization, and tests. e-learning: electronic learning.

The experiment was conducted from November 2018 to December 2018. The flow of the experiment is shown in Figure 2. The 44 eligible volunteers were asked to complete the pretest in 30 min, and the unqualified participants were eliminated based on the results. The randomization sequence was computer generated using IBM SPSS version 24. Then, the qualified participants were randomly divided into two groups: the e-learning group (EG) and the non–e-learning control group (CG). The enrolled students spent 1 month in theoretical and practical training for gastroscopy examinations in our on-campus endoscopy center (Endoscopy Center, the Second Affiliated Hospital of HMU). A specific username and password were assigned to each EG participant to log in to the GRS during the trial. The EG had access to traditional learning combined with e-learning at any time and location. During the same period, the CG did not have access to the e-learning tool and learned only through the traditional mode of reading textbooks and other written materials in addition to practical training. All participants received routine gastroscopy training at the endoscopy center during the trial.

After the 1-month gastroscopy study, all participants were required to complete the posttest and gastroscopy examination assessment. The EG also completed an additional questionnaire to determine the level of satisfaction with the GRS.

### Learning Outcome Evaluation Strategy

The potential and advantages of e-learning may not always be perceived as leading to significant improvements in educational outcomes. Therefore, it is necessary to demonstrate the rationality of e-learning through the assessments of effectiveness and efficacy. Kirkpatrick built a famous framework to evaluate the learning effect in the 1950s [27], which can be used to assess GRS effectiveness [24,28,29]. The framework describes four assessment levels: reaction, learning, behavior, and results [19,30]. Most studies that evaluate e-learning processes rely only on users’ learning and reaction rather than behavior and results [21,30-33]. GRS was designed to help improve the gastroscopic practice skills of primary learners. On this basis, e-learning in this study was further evaluated at the *behavior* level to reveal the efficiency and reliability of e-learning in medical education.

### Pre- and Postintervention Tests

For the *learning* level assessment, the most conventional and reasonable test method is the comparison of pre- and postintervention test scores. In our experiment, the participants completed a pretest before gastroscopy learning and a posttest after 1 month of training. The pretest and posttest contained the same set of questions, but the order of the questions differed to ensure that the difficulty and reference standards of the two tests were at the same level [23,34]. The tests consisted of 50 multiple-choice questions, each of which had 4 to 5 response options and a single correct answer. Each question had a value of two points, with a total possible score of 100 points. The main topics of the multiple-choice questions were the elements of the basic operation of gastroscopy, cognition of the anatomical structure of the UGI, mastery of the characteristics of the structure of the UGI under gastroscopy, and the identification of and distinction between images of various common diseases of the UGI under gastroscopy. Most endoscopic images used in the questions were taken from the linked pictures used in the GRS system, and the rest were from our endoscopy center. The test was compiled by the authors of this paper and the designer of the GRS software. In total, 2 endoscopy experts from the Department of Gastroenterology, the Second Affiliated Hospital of HMU, reviewed the test questions to ensure that they were suitable for this experiment. The test paper (pretest version) is provided as Multimedia Appendix 1.

To ensure the fairness of the test and the reliability of the scores, we placed the participants in a classroom with a signal shield and conducted a closed-volume test with 2 invigilators and timers. The examination time was set at 30 min. When the time ended, the participants were asked to stop, and the papers were collected immediately. After the pretest, the participants could not see their own answers or the correct answers to ensure the validity of the posttest.

### Gastroscopy Examination Test

As our experiment was based on the combination of e-learning and traditional endoscopy teaching (ie, blended learning), we tested the participants’ actual gastroscopy examination performance to evaluate the *behavior* level. After 1 month of training, the students undertook a gastroscopy examination, with the examination order based on drawing lots, and made real-time video recordings. All the patients participating in the gastroscopy test had taken an appointment for a general gastroscopy at the outpatient department and had agreed to a gastroscopy performed by primary endoscopists. As many unexpected problems appeared in the actual operation, such as patients with a special physiological structure or patients with lesions who were difficult to identify, teachers were present during the procedure. Each participant had three opportunities to select the performance that he or she thought was the most satisfactory for archiving. A total of 5 expert endoscopists (from the Affiliated Hospital of HMU) who were not aware of the participants’ grouping scored the students’ gastroscopy examination videos based on their experience in gastroscopy. The evaluation standard applied in the examination was the scale developed jointly by experts, including forward operation of gastroscopy, withdrawal observation, patients’ comfort and satisfaction, overall gastroscopy examination time and fluency, clarity of collected images, and diagnostic accuracy. We removed the highest score and the lowest score and obtained the average final score. The grading table is provided as Multimedia Appendix 2.

### Gastroscope Roaming System Satisfaction Questionnaire

The *reaction* level was assessed by a satisfaction questionnaire. We used the questionnaire developed by Wang [35,36], which has been widely cited in evaluations of e-learning satisfaction [23]. This questionnaire has been shown to reach a reliability (Cronbach alpha) of .95 [35]. As the current version of the GRS is asynchronous, we did not use the *learning community* quality of Wang’s questionnaire. With the exception of the last two questions about overall satisfaction and success, we divided the remaining 22 questions into four modules: content, interface, testing, and personalization. We translated the questionnaire into Chinese and used a 5-point Likert scale to estimate each question anchored with *strongly disagree* to *strongly agree* and then counted the total points [37]. All the students in the EG completed the satisfaction questionnaire after completing their study. The questionnaire is provided as Multimedia Appendix 3.

### Statistical Analysis

IBM SPSS version 24 was used for data analysis. The analysis included simple frequencies and descriptive analyses (means and standard deviations). First, the interaction of variables was verified by a general linear model. A paired sample *t* test was used to compare the test scores before and after the intervention in each group, and an independent sample *t* test was used to compare the pretest, posttest, and gastroscopy examination scores between the EG and the CG. The differences in the sex ratio and grade composition of the participants were analyzed by the chi-square test. For all the statistical analyses, we considered the significance level to be .05. Data analysis was used to determine whether there was a significant difference between the two experimental groups.

## Results

### Sample Description

Of the 44 masters degree students from the Second Affiliated Hospital of HMU who were assessed for eligibility, 42 (95%) who met the inclusion criteria completed the pretest and were included in the study. Two participants were excluded because one failed to complete the pretest and the other had a pretest score that was too high (>90%). Of the remaining participants, 21 were assigned to the EG and 21 to the CG following the principles of randomized controlled trials. During the learning period, only 1 student in the EG dropped out of the course. Therefore, data for 20 students in the EG and 21 students in the CG were analyzed. All 20 of the remaining students in the EG completed the satisfaction questionnaire. The baseline characteristics were similar in both the groups. Table 1 summarizes the characteristics of the participants.

**Table 1:** Demographic and other characteristics of participants.

Baseline characteristics		Electronic learning group (n=20)	Control group (n=21)	*P* value
**Age** **(years)**				.77
	Range	23-27	23-26	
	Mean	24.05	24.14	
**Gender, n (%)**				.39
	Male	6 (30)	9 (43)	
	Female	14 (70)	12 (57)	
**Current year of residency training, n (%)**				.44
	Postgraduate year 1	12 (60)	15 (71)	
	Postgraduate year 2	8 (40)	6 (29)	
**Pretest score**				.96
	Range	40-60	38-62	
	Mean	50.86	50.76	

### Statistics and Analysis of Examination Scores

The mean pretest score (SD) in the EG was 50.86 (SD 6.11), and it improved to 83.70 (SD 5.99) in the posttest (*P*<.001). In contrast, the mean pretest score (SD) in the CG was 50.76 (SD 6.88), and it increased to 78.76 (SD 7.58) in the posttest (*P*<.001). After using the general linear model to analyze the difference between the EG and CG in the pretest and posttest, no interaction was found between the between-group variable and the within-group variable (*P*=.11). Therefore, a *t* test was used for further analysis. The results showed that there were no significant differences between the EG and CG (*P*=.96) in the pretest. After 1 month of learning, the posttest results of the EG were better than those of the CG (*P*=.03), and the test scores of the EG improved more than those of the CG (*P*=.01).

Regarding the gastroscopy examination scores, the mean score (SD) was 91.05 (SD 4.58) in the EG and 84.38 (SD 5.19) in the CG (*P*<.001).

### User Satisfaction With the Gastroscope Roaming System

**Figure 3:**
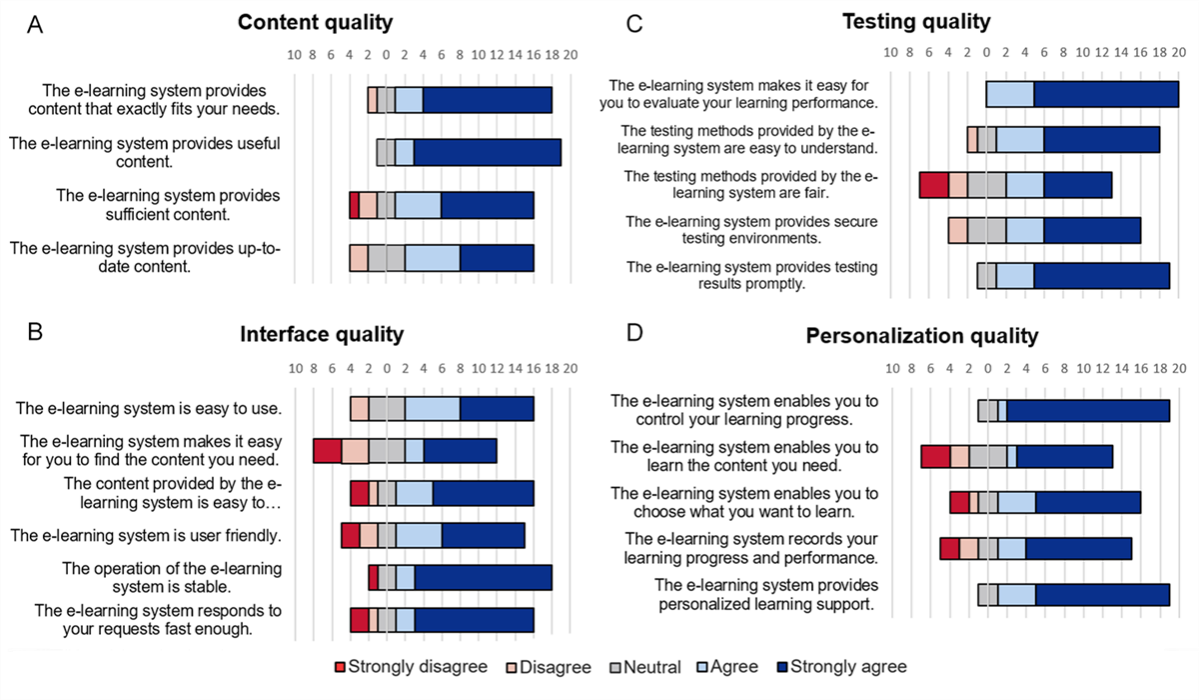
Satisfaction analysis of the system by Gastroscope Roaming System participants in four modules: (A) content quality, (B) interface quality, (C) testing quality, and (D) personalization quality. Each module lists the problems it contains. e-learning: electronic learning.

The overall satisfaction rate with the e-learning course was 77.0% (308/400). Only 11.5% (46/400) of the students were biased against e-learning, and the remaining 11.5% (46/400) maintained a neutral attitude. The last two overall satisfaction problems showed that 85% (17/20) of the participants were satisfied with the GRS, and 95% (19/20) of the participants thought it was successful. The satisfaction level for each problem is detailed in Figure 3.

Regarding the content quality (Question 1-Question 4), 80% (64/80) of the learners thought that the course fit their needs and was sufficient, useful, and up-to-date. Regarding the interface quality (Question 5- Question 10), 73.3% (88/120) of the participants found the e-learning course to be stable, user friendly, and fast and agreed that it was easy to find the content needed. Regarding the testing quality (Question 11-Question 15), 80% (80/100) of the learners assessed the testing of the course as fair, secure, prompt, easy to understand, and easy to evaluate. Regarding the personalization quality (Question 16-Question 20), 76% (76/100) of the students thought that the GRS enabled them to learn the content needed, choose what to learn, control their learning progress, record their learning progress, and provide personalized learning support. Hence, there were more oppositional responses to the interface and personalization qualities than to the other qualities.

## Discussion

### Principal Findings

The first main outcome of this study was the development of an e-learning software named *Gastroscope Roaming System* for primary learners. To date, the conventional gastroscopy teaching mode has been face-to-face tutorials. The number of learners is, therefore, very small; the instruction is lengthy; and it is not convenient to practice only on patients. Textbooks and handouts appear to be the extensive way of disseminating knowledge in education [38]. However, for students in the field of medical operations, especially endoscopy teaching, traditional lectures and spectrograms are not conducive to learning how to operate and gain experience effectively. In addition, gastroscopy simulators have problems of high cost, limited teaching resources, and low popularity. As constructed in this experiment, an internet-based e-learning system offers tremendous advantages over the traditional teaching methods because there are no limits on the number of learners or learning time and place. Students can study gastroscopy with their laptop at any time and place instead of being confined to an observational study in the narrow gastroscopy room, thus enabling them to more reasonably allocate and use their study time. Gastroscopy learning has three aspects: knowledge, skills, and experience. Through conventional lectures or hands-on seminars, endoscopists can acquire only the relevant knowledge and techniques [39]. Therefore, we collected many endoscopic images of common UGI diseases and inserted them into the software for primary learners to accumulate experience. Given the creation of the new gastroscope learning method, we can expect to apply this method to more endoscopic learning and training.

The second principal result was the comparison of the learning results of the GRS-based EG with those of the traditional learning group, showing that e-learning can improve the learning efficiency of primary gastroscopists. Notably, the EG in this experiment used a combination of e-learning and traditional learning, that is, blended learning, which seemed to be more effective than single e-learning with respect to knowledge acquisition. Although e-learning may not be an alternative to traditional learning in some cases, it can provide supplements to traditional education and is a useful complement to traditional education [40-42].

Before gastroscopy learning, there was no significant difference between the two groups in the predicted test, indicating that the level of the participants was similar. After 1 month of study, the theoretical test scores of all the students significantly improved, and the score of the EG increased slightly more than that of the CG. However, the gastroscopy examination score of the EG was notably higher than that of the CG after 1 month of learning, indicating that e-learning is more effective than traditional learning, especially in improving students’ gastroscopic handling ability. The theoretical-level test verified the participants’ basic knowledge of gastroscopy and their ability to diagnose disease, whereas the gastroscopy examination verified their practical ability. In other words, gastroscopy ability=cognitive ability+practical ability. The technical level is most important for beginners learning gastroscopy. Therefore, we believe that the GRS is essential for helping students master gastroscopy skills in a short time. The GRS provides learners with a virtual UGI space, which enables them to explore the structure of the UGI freely and then, through repeated learning, to master and familiarize themselves with the structure of the UGI. In the process of a real gastroscopy procedure, they can operate skillfully and confidently, accelerate their learning speed, and avoid the harm caused to patients by confusion and fear. Moreover, by repeatedly studying the endoscopic pictures and characteristics of common diseases shown in the GRS, students can learn to accurately identify lesions in actual gastroscopy.

The third main result was that the satisfaction questionnaire showed that the overall satisfaction rate of the students in the EG was very high. Using Wang’s questionnaire, we determined how satisfied the students were with each part. We found that the students were more satisfied with the content and testing qualities and less satisfied with the interface and personalization qualities. In terms of the interface quality, students found that the operational interface of the software was somewhat complex, difficult to understand, and not smooth. This response may be because of the software design using the *W/A/S/D* keys on keyboard with mouse movement. The students had different proficiency levels in computer operation and different computer configurations.

### Limitations

Our study also has limitations. For example, the small sample size in a single institution affects the generalizability of the results. The main reason was that our GRS software was in the early stage of construction. We will continue to improve, upgrade, and expand its content in the future. Therefore, the software was applied only on a small scale in the hospital with which the author is affiliated to assess the efficiency and effectiveness of the e-learning model. Regarding the assessment of the real gastroscopy procedure, because of differences in the physiological structure of the UGI and the pain and tolerance levels of each patient, it was not guaranteed that each student would face the same difficulty in the assessment. Even with three opportunities, the participants might not have been able to perform at the real level of gastroscopy, although the probability of this problem is low. Finally, we should carefully consider the validity of the satisfaction assessment. We used an unproven Chinese version of the user satisfaction questionnaire because we could not find a Chinese-validated translation for e-learning systems in the literature.

### Conclusions

The results of our study suggest that e-learning is an effective educational strategy for primary learners to acquire skills in gastroscopy examination and master the characteristics of endoscopic images of the UGI. The GRS may serve as a meaningful supplementary approach to on-campus endoscopy education. The novel computer assistant module in the field of endoscopic teaching will strengthen the training of skilled endoscopists. We will continue to upgrade and improve the GRS system to enhance the authenticity of the simulation and to enrich the variety of diseases. In summary, e-learning can be widely used as an effective assistant to supplement routine teaching and learning in medical education.

## Appendix

Multimedia Appendix 1 Multiple-choice question test paper for evaluating Gastroscope Roaming System learning efficiency.

Multimedia Appendix 2 Grading table for gastroscopy examination.

Multimedia Appendix 3 Gastroscope Roaming System satisfaction questionnaire.

Multimedia Appendix 4 CONSORT-EHEALTH (V 1.6.1) -Submission/Publication Form.

## Abbreviations

CG: non–e-learning control group
CMB: China Medical Board
e-learning: electronic learning
EG: e-learning group
GI: gastrointestinal
GRS: Gastroscope Roaming System
HMU: Harbin Medical University
UGI: upper gastrointestinal
