# Supplementary material for: Development and Assessment of a Gastroscopy Electronic Learning System for Primary Learners: Randomized Controlled Trial
Source: J Med Internet Res. 2020 Mar 23;22(3):e16233. doi: 10.2196/16233 (PMC7136842; doi:10.2196/16233)
Supplement: Multimedia Appendix 1 [file jmir_v22i3e16233_app1.pdf]

“Gastroscope Roaming System” Learning Efficiency Test

(Candidates need to complete the test within 30 minutes)

(Proposer: Shizhu Jin, Shuang Li, Department of Gastroenterology, Second Affiliated Hospital of Harbin Medical University)

Candidate name:_______________ **Test time:**__________________ **Score:** ______________

Multiple choice questions (each question has only one correct answer. 2 points for each question, a total of 50 questions, out of 100 points.)

1. Which of the following patients can be examined by gastroscopy? ( )

A. Mental disorders

B. Severe coagulation disorders

C. The patient himself refused to sign the informed consent for gastroscopy

D. Severe cardiopulmonary, hepatopulmonary insufficiency

E. Upper gastrointestinal bleeding

2. For routine gastroscopy, the most suitable position for patients is ( )

A. Left lateral recumbent position with flexion of both knees
B. Right lateral decubitus with flexion of both knees
C. Supine position, knees straight
D. Left recumbent position with knees straight
E. Prone position, knees straight

3. As shown in the gastroscopic image, the position of the gastroscope at this time is approximately which of the following sketches ( )

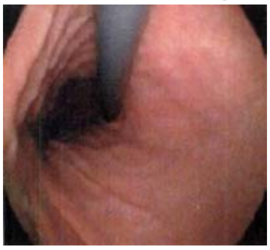

A. 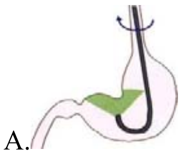 B. 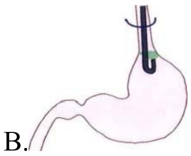 C. 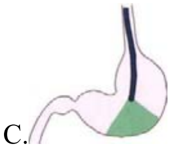 D. 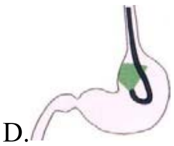 E. 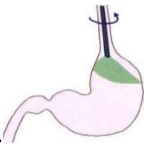

4. Which upper digestive tract structure is shown in the figure under gastroscopy ( )

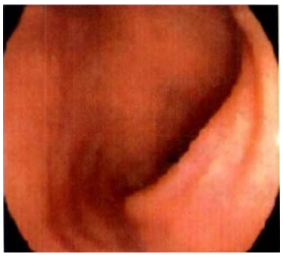

A. Gastric body B. Gastric angle C. Duodenal descending D. Duodenal bulb E. Cardia

5. The three physiological strictures of the normal adult esophagus are about ( )

A. 10cm，15cm， 20cm

B. 15cm，25cm，40cm

C. 15cm，25cm，35cm

D. 10cm，25cm，40cm

E. 20cm，30cm，40cm

6. Which segment of the upper digestive tract is shown under gastroscopy?( )

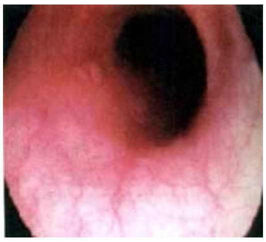

A. upper esophagus B. middle esophagus C. lower esophagus D. cardia E. pylorus

7. According to the gastroscopic image in the following picture, it can be diagnosed as ( )

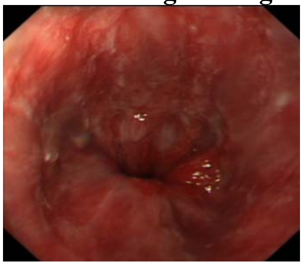

A. Reflux esophagitis B. Chronic superficial gastritis C. Duodenal glomerulitis
D. Normal esophagus E. Cardiac achalasia

8. The features of grade C endoscopic images of reflux esophagitis (Los Angeles) are ( ).

A. At least one fusion injury with more than two mucosal folds
B. The length of mucosal lesions should not exceed 5 mm.
C. Whole-week mucosal injury
D. At least one mucosal lesion was more than 5 mm in diameter, but no fusion occurred.
E. Mucosal lesions are interconnected, exceeding 3/4 of the circumference of the esophagus.

9. Which of the following endoscopic images can be diagnosed as esophageal papilloma ( )

A. 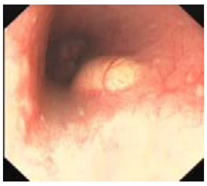 B. 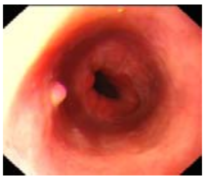 C. 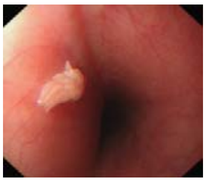 D. 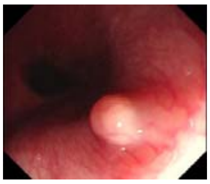

10. In the following endoscopic picture, chronic atrophic gastritis is ( )

A. 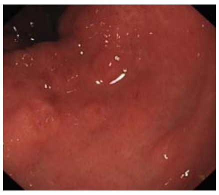 B. 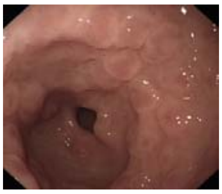 C. 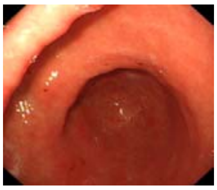 D. 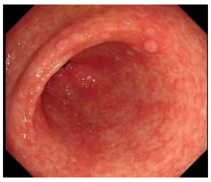

11. The most common site of gastric ulcer is ( )

A. Fundus B. Antrum C. Greater Curvature and Angle D. Smaller Curvature and Antrum E. Angle and Smaller Curvature

12. Endoscopic features of gastric ulcer inactive stage (A1 stage) are ( )

A. 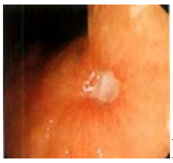 B. 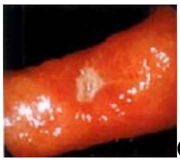 C. 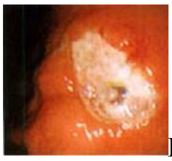 D. 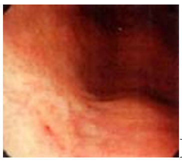 E. 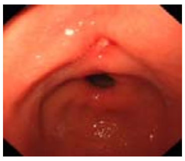

13. Which of the following two groups of gastroscopic pictures is benign ulcer ( )

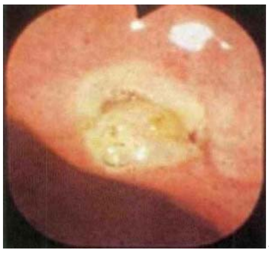
a

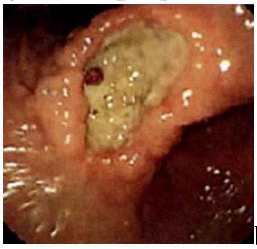
b

A. All are benign ulcers
B. A is, B is not.
C. A is not, B is
D. None of them are benign ulcers
E. Uncertainty of judgment

14. In the following endoscopic images of gastric polyps, Yamada type III is ( )

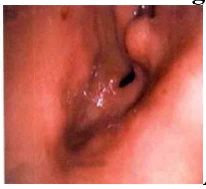
A
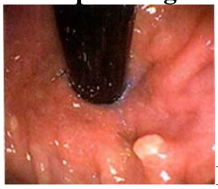
B
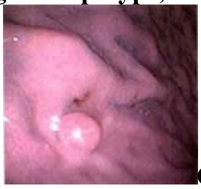
C
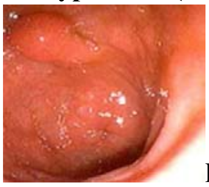
D

15. Endoscopic staging of early gastric cancer (Japanese Endoscopic Classification) is ( )

A. Uplift type, superficial type, and depression type
B. Superficial, protruding and infiltrating types
C. Surface type, infiltrating type, and mixed type
D. Surface type, fungus type, and depression type
E. Protuberant, superficial and infiltrative type

16. The endoscopic image of advanced gastric cancer shown below is ( ) according to Borrmann classification of gastric cancer.

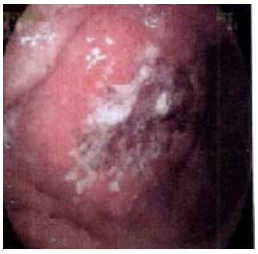

A. type 1 – raised
B. type 2 – ulcer
C. type 3 – ulcerative infiltrating
D. type 4 – diffuse infiltrating
E. type 5 – unshaped

17. Which of the following is characteristic of stage H1 duodenal ulcer ( )

A. The bottom is covered with thick moss, and there are hemorrhagic spots or clots attached. The surrounding mucosa is markedly hyperemic, edematous and erosive.

B. Peripheral hyperemia and edema of ulcer were alleviated, white moss was clean, the boundary was clear, and the concentration of mucosal folds was not obvious.

C. The ulcer shrank and became shallow, the white fur edge was smooth, the edema disappeared, the surrounding regenerated epithelium was obvious, and the mucosal fold concentrated sign appeared.

D. The ulcer was reduced, the white fur became thinner and the regenerated epithelium was widened.

E. All ulcers are covered by regenerated epithelium, which is red and arranged in grid-like centripetal radiation.

18. Here is a gastroscopic picture, which can be diagnosed as（ ）

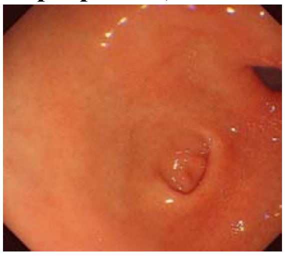

A. gastric ulcer B. gastric Vater ampulla pancreas C. concave gastric cancer D. gastric submucosal tumor E. duodenal Vater ampulla pancreas

19. The characteristics of gastroscopic images of duodenal polyps are as follows（ ）

A. Round or irregular protuberances with peduncles or peduncles mostly non-smooth surfaces, with fissures or lobulations
B. Protuberances of varying sizes with hyperemia, erosion, and bleeding on the surface
C. Isolated, encapsulated masses, usually small and peduncle less, with bluish or red color and clear demarcation from the surrounding mucosa
D. Submucosal bulge with yellowish-white color, soft texture, round or lobulated
E. Soft texture, transparency, drainage of cystic fluid after biopsy and reduction of volume

20. The diagnosis of the disease in the gastroscopic picture below is（ ）

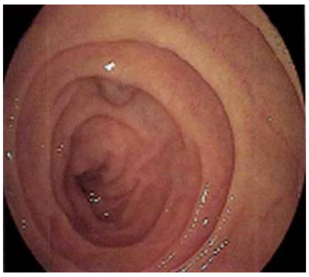

A. Duodenal leiomyoma B. Duodenal hemangioma C. Duodenal Brunner adenoma

D. Duodenal polyps E. Duodenal venous aneurysms

21. When patients undergo general gastroscopy, the correct angle of head and neck is（ ）

A. 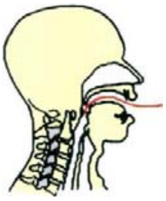 B. 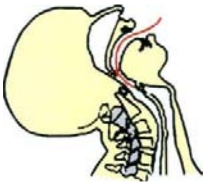 C. 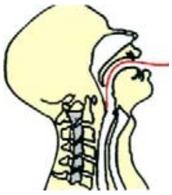

D. ABC is correct E. ABC is incorrect.

22. Before general endoscopy, the following statements are incorrect（ ）

A. Confirmation of the monitoring screen, endoscopic ventilation, and water supply without fault
B. Left-handed endoscopy operating unit with a thumb to adjust upper and lower, left and right knobs
C. The right-hand holds the mirror body with a pen, and the hand position is about 25 cm away from the end of the mirror.
D. Apply silicone oil or lubricating painkiller to the lens
E. Assist the patient to set up his position and ask the patient to relax and cooperate with the examination.

23. The following pictures are in accordance with the endoscopic grading of reflux esophagitis (Los Angeles) D-level endoscopic image characteristics（ ）

A. 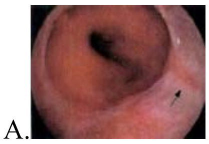 B. 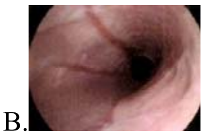 C. 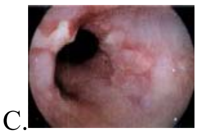 D. 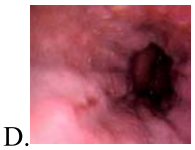 E. 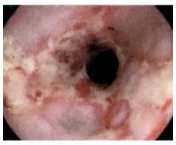

24. In the following endoscopic images, Mallory-Weiss syndrome can be diagnosed as（　）

A. 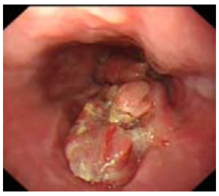 B. 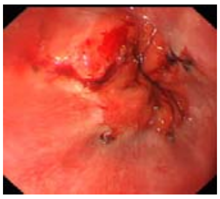 C. 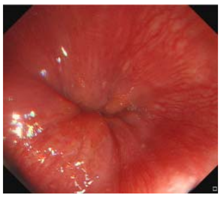 D. 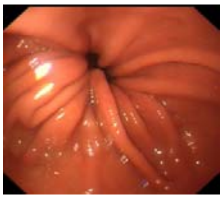

25. In the following gastroscopic images, the diagnosis of gastric lipoma is（　）

A, 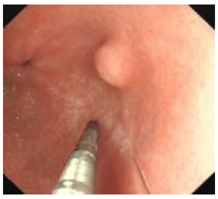 B. 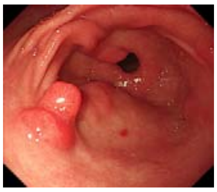 C. 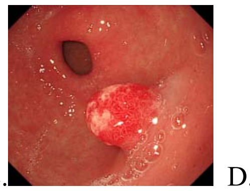 D. 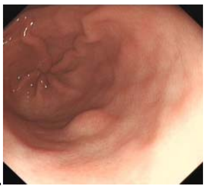

26. Regarding preoperative preparation for gastroscopy, the following statements are incorrect（ ）

A. Start fasting 12 hours before the inspection and drink water.

B. Absolutely abstain from drinking water and take antihypertensive drugs after going to bed on the day of inspection.

C. To inhibit gastric peristalsis and secretion of gastric juice, antispasmodics and sedatives can be selected 30 minutes before examination according to the situation.

D. Patients were given lidocaine glue 30 minutes before operation

E. If the patient has a removable denture, it should be removed.

27. In the course of gastroscopy, if an improper operation results in the reflex of gastroscopy in the esophagus, the following is correct（ ）

A. Pull out the mirror immediately

B. Remove reflex immediately in the esophagus

C. Fast insertion into the stomach to relieve reflex in a wide stomach cavity

D. Slowly introduce a curved mirror body into the stomach to relieve reflex in a wide stomach cavity

E. Quickly panicked to leave

28. The second esophageal stricture is located（ ）

A. Aortic arch and tracheal bifurcation B. Upper sternal margin C. Ascending aorta and organ bifurcation D. Cyclothyroidism Level　E. About 30 cm from the incisor

29. The following gastroscopic image is most likely to be diagnosed（ ）

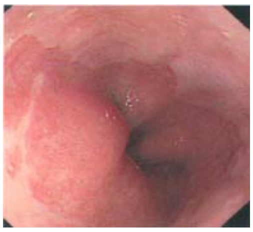

A. Reflux esophagitis B. Esophageal ectopic gastric mucosa C. Barrett esophagus

D. Behcet's disease　E. Esophageal glycogen acanthosis

30. The following figure can be diagnosed as（ ）

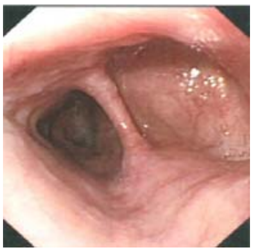

A. Esophageal polyp B. Cardiac achalasia C. Barrett esophagus D. Esophageal hiatal hernia

E. Esophageal diverticulum

31. The most likely diagnosis of achalasia in the following figure is（ ）

A. 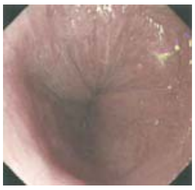 B. 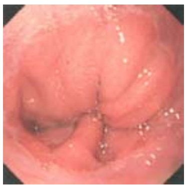 C. 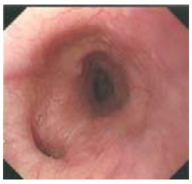 D. 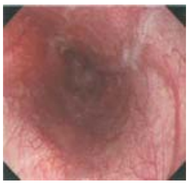

32. The following figure can be diagnosed as（ ）

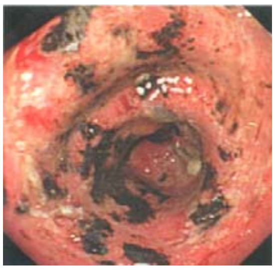

A. Gastrolithiasis B. Chronic atrophic gastritis C. Pox-like gastritis D. Acute erosive hemorrhagic gastritis E. Behcet's disease

33. The following figure can be diagnosed as（ ）

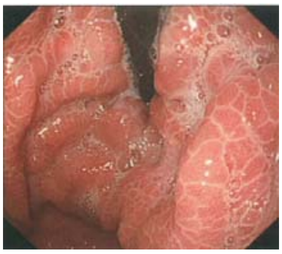

A. acute gastritis B. portal hypertension gastropathy C. gastric cancer D. gastric ulcer

E. multiple gastric polyps

34. This is not an upper gastrointestinal hemangioma in the following gastroscopy（ ）

A. 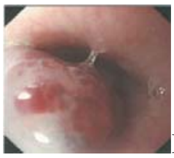B. 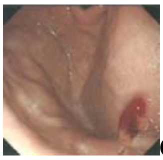C, 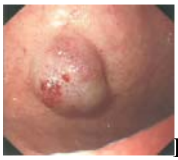D. 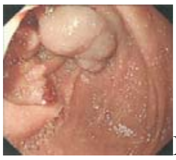E. 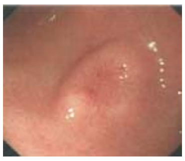

35. The diagnosable diseases in the figure below are（ ）

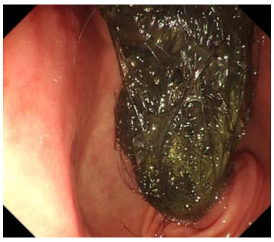

A. gastric cancer B. gastric ulcer C. gastric cyst D. gastric xanthoma E. gastrolithiasis

36. The following figure can be diagnosed as（ ）

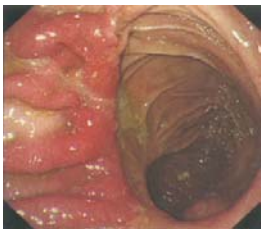

A. gastric ulcer B. duodenal ulcer C. gastritis D. gastric cancer E. duodenal tumor

37.   The figure below is the most likely diagnosis（ ）

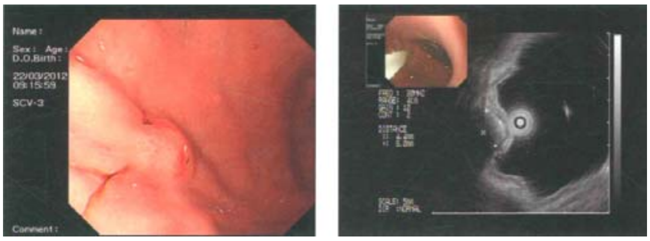

A. Gastric lipoma B. Gastric leiomyoma C. Gastric polyp D. Gastric cyst E. Gastric cancer

38. The following figure can be diagnosed as（ ）

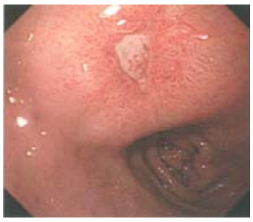

A. Gastric ulcer B. Duodenal bulbar ulcer C. Duodenal descending ulcer D. Duodenal tumor E. Malignant gastric ulcer

39. The most likely diagnosis is shown in the figure below（ ）

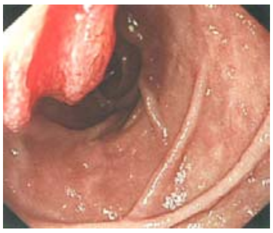

A. Duodenal lymphoma   B. Duodenal lipoma   C. Duodenal hemangioma

D. Duodenal papilloma   E. Duodenal papillary cancer

40. The following figure is most likely to be diagnosed as（ ）

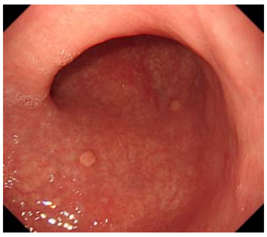

A. Gastric polyps B. Gastric hemangioma C. Gastric heterotopic pancreas D. Gastric xanthoma E. Gastric lipoma

41. The following figure can be diagnosed as（ ）

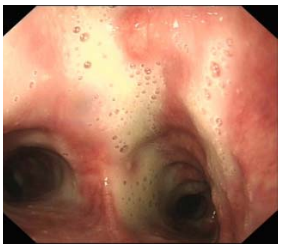

A. Postoperative gastric B. Duodenal diverticulum C. Esophageal diverticulum D. Esophageal-tracheal fistula E. Cardiac achalasia

42. The following figure can be diagnosed as（ ）

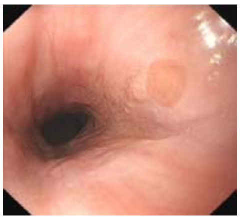

A. Heterotopic gastric mucosa of esophagus B. Barrett esophagus C. Reflux esophagitis D. Fungal esophagitis E. Esophageal glycogen acanthosis

43. Which of the following is not an esophageal glycogen acanthosis （ ）

A. 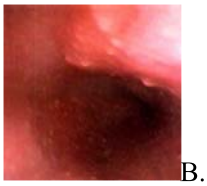 B. 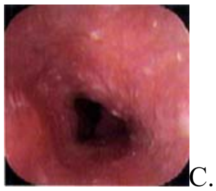 C. 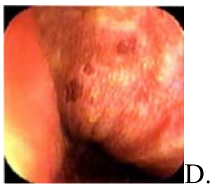 D. 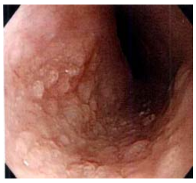

44. Which can be diagnosed as intestinal metaplasia in the given figures（ ）

A. 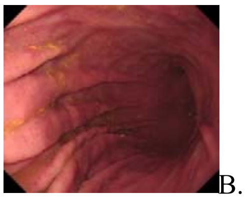 B. 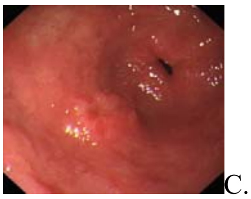 C. 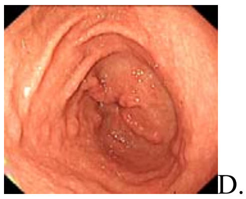 D. 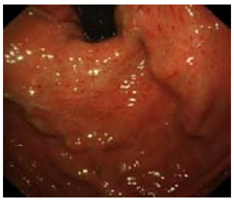

45. The following figure can be diagnosed as（ ）

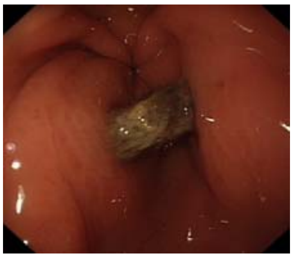

A. Gastric cancer B. Gastric papilloma C. Gastric foreign body D. Gastric lithiasis E. Gastric ulcer

46. The following figure is most likely to be diagnosed as（ ）

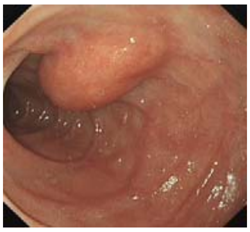

A. Duodenal lipoma B. Duodenal hemangioma C. Duodenal papilloma

D. Duodenal Brunner adenoma E. Duodenal cyst

47. Duodenal inflammation is not seen in which of the following figure（ ）

A. 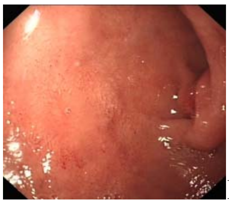 B. 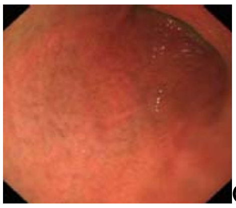 C. 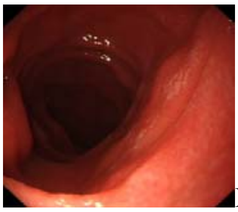 D. 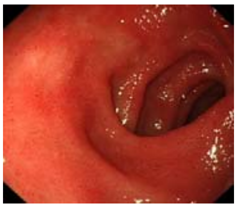

48. The following figure can be diagnosed as（ ）

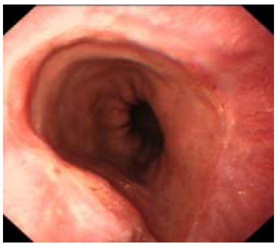

A. Achalasia B. Reflux esophagitis C. Esophageal and cardiac mucosal laceration D. Esophageal hiatal hernia E. Esophageal diverticulum

49. The following figure can be diagnosed as（ ）

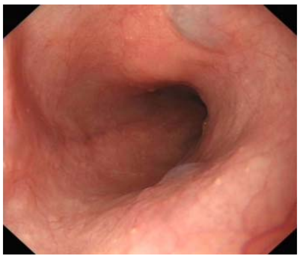

A. Esophageal polyps B. Esophageal varices C. Esophageal leiomyoma D. Esophageal papilloma E. Esophageal venous aneurysm

50. The following figure is most likely to be diagnosed as（ ）

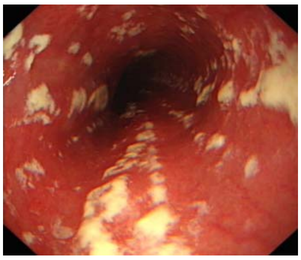

A. Reflux esophagitis B. Esophageal glycogen acanthosis C. Esophageal foreign body

D. Esophageal cancer E. Fungal esophagitis
