# Supplementary material for: Development and Assessment of a Gastroscopy Electronic Learning System for Primary Learners: Randomized Controlled Trial
Source: J Med Internet Res. 2020 Mar 23;22(3):e16233. doi: 10.2196/16233 (PMC7136842; doi:10.2196/16233)
Supplement: Multimedia Appendix 2 [file jmir_v22i3e16233_app2.pdf]

Grading Table for Gastroscopy Examination

Candidate number：___________　　Score：____________

Examination contents		Fraction	Score
1.Gastroscop forward operation	Preparation before the examination, holding gastroscope method	5	
	Operation from oropharyngeal to esophageal entrance	5	
	Operation through esophagus	5	
	Entering the body of the stomach through the cardia and fundus of the stomach	5	
	Operation through gastric body and antrum	5	
	Access to the duodenum through the pylorus	5	
	Entering the descending segment of the duodenum through the upper corner of the duodenum	5	
2.Gastroscopy withdrawal observation	Observation of descending segment of duodenum	5	
	Observation of duodenal bulb	5	
	Observation of gastric antrum	5	
	Observation of gastric angle	5	
	Observation on the fundus and cardia of stomach	5	
	Observation of gastric body	5	
	Observation of esophagus and cardia	5	
3. Overall gastroscopy examination time and fluency		5	
4. Response, comfort and satisfaction of patients during gastroscopy		5	
5. Position and definition of collected image		10	
6. Diagnostic accuracy under gastroscope		10	
Total		100	
Errors or deficiencies of candidates:			
Signature of assessment expert:	Date:		
