# Supplementary material for: Development and Assessment of a Gastroscopy Electronic Learning System for Primary Learners: Randomized Controlled Trial
Source: J Med Internet Res. 2020 Mar 23;22(3):e16233. doi: 10.2196/16233 (PMC7136842; doi:10.2196/16233)
Supplement: Multimedia Appendix 3 [file jmir_v22i3e16233_app3.pdf]

“Gastroscope Roaming System” Satisfaction Questionnaire

In order to evaluate the learning efficiency of "Gastroscope Roaming System" software and to improve the software's shortcomings, please give a real and objective feedback after using the GRS, Please mark the selected items. Thank you for your cooperation!

Satisfaction Score: Strongly agree/Agree/Neutral/Disagree/Strongly disagree

**Number**	**Survey Items**	**Survey Content**		**Satisfaction Score**				
				Strongly agree	Agree	Neutral	Disagree	Strongly disagree
**A**	**Content quality**	Q1	The e-learning system provides content that exactly fits your needs.					
		Q2	The e-learning system provides useful content.					
		Q3	The e-learning system provides sufficient content.					
		Q4	The e-learning system provides up-to-date content.					
**B**	**Interface quality**	Q5	The e-learning system is easy to use.					
		Q6	The e-learning system makes it easy for you to find the content you need.					
		Q7	The content provided by the e-learning system is easy to understand.					
		Q8	The e-learning system is user-friendly.					
		Q9	The operation of the e-learning system is stable.					
		Q10	The e-learning system responds to your requests fast enough.					
**C**	**Testing quality**	Q11	The e-learning system makes it easy for you to evaluate your learning performance.					
		Q12	The testing methods provided by the e-learning system are easy to understand.					
		Q13	The testing methods provided by the e-learning system are fair.					
		Q14	The e-learning system provides secure testing environments.					
		Q15	The e-learning system provides testing results promptly.					

**D**	**Personalization quality**	Q16	The e-learning system enables you to control your learning progress.					
		Q17	The e-learning system enables you to learn the content you need.					
		Q18	The e-learning system enables you to choose what you want to learn.					
		Q19	The e-learning system records your learning progress and performance.					
		Q20	The e-learning system provides the personalized learning support.					
**E**	**Overall quality**	Q21	As a whole, you are satisfied with the e-learning system.					
		Q22	As a whole, the e-learning system is successful.					
**Other comments:**								
